# ECMO in the Cardiac Catheterization Lab—Patient Selection Is Key

**DOI:** 10.3390/jcdd12010012

**Published:** 2024-12-31

**Authors:** William Tracy, Brandon E. Ferrell, John P. Skendelas, Mayuko Uehara, Tadahisa Sugiura

**Affiliations:** 1 Department of General Surgery, NYC Health + Hospitals/Metropolitan, New York, NY 10029, USA; tracyw@nychhc.org; 2 Department of Cardiothoracic and Vascular Surgery, Montefiore Medical Center, Bronx, NY 10467, USA; bferrell@montefiore.org (B.E.F.); jskendel@montefiore.org (J.P.S.); muehara@montefiore.org (M.U.)

**Keywords:** extracorporeal membrane oxygenation, percutaneous coronary intervention, transcatheter aortic valve replacement, catheter ablation, cardiac catheterization, extracorporeal cardiopulmonary resuscitation, cardiac arrest

## Abstract

The use of extracorporeal membrane oxygenation (ECMO) has emerged as a rescue intervention for hemodynamically unstable patients and prophylactic intraprocedural hemodynamic support in the cardiac catheterization laboratory. The prompt initiation of ECMO provides immediate hemodynamic support and allows for the completion of bridging and/or life-saving interventions. However, there are no clinical practice guidelines for the use of extracorporeal support in this area. This review examines the role of patient selection and therapeutic intervention for extracorporeal support in the cardiac catheterization laboratory.

## 1. Background

Veno-arterial extracorporeal membrane oxygenation (V-A ECMO) is a crucial and life-preserving adjunct of care to the critically ill patient in refractory cardiogenic shock. ECMO is commonly initiated in the intensive care unit for hemodynamically unstable patients, the operating room for post-cardiotomy shock, or as extracorporeal cardiopulmonary resuscitation (ECPR) in cardiac arrest. In contrast, the cardiac catheterization laboratory (CCL) is a procedural area in which percutaneous coronary, structural heart, and electrophysiology interventions may be performed with or without surgical capabilities (i.e., hybrid operating rooms). Patients in these settings range from elective outpatient procedures to those in refractory cardiogenic shock. Access to mechanical circulatory support in the CCL to bridge or rescue patients in cardiac arrest or refractory heart failure can vary. Furthermore, CCLs are often insufficiently staffed and poorly equipped to resuscitate patients once ECMO is initiated. These patients require critical care management for ongoing resuscitation with the risk of direct patient harm if not managed appropriately.

Current recommendations from The American College of Cardiology/American Heart Association guidelines recommend intra-aortic balloon pump (IABP) use in patients with acute myocardial infarction (AMI) and cardiogenic shock (CS) with a class IIa (level of evidence B) recommendation, while the European Society of Cardiology/European Association for Cardio-Thoracic Surgery guidelines give it a class III (level of evidence A) recommendation. Mechanical circulatory support devices, e.g., Impella (Abiomed, Danvers, MA, USA) and TandemHeart (LivaNova, London, UK), provide superior hemodynamic support compared to IABP, but early studies did not identify differences in 30-day all-cause mortality [1,2]. Recent studies on the Impella CP, with a standardized approach and early initiation, have shown a 76% hospital survival rate in AMI and CS patients, which is higher than the 50–60% seen in earlier reports [3,4]. However, these devices may be insufficient for patients with severe CS, biventricular failure, or refractory cardiac arrest. In such cases, V-A ECMO can provide full cardiopulmonary support (>4.5 L/min) independent of unstable arrhythmias.

In accordance with recommendations like those previously stated, the general use of ECMO has been gaining favor in the past 20 years with an estimated 50-fold increase in utilization [5]. The growing use of ECMO has also expanded into other facets of cardiac care, including in the CCL. This means that CCL providers and staff should be aware of the absolute and relative contraindications for ECMO in preparation for potential patients. Knowing this information, CCL providers should formulate an “ECMO Activation Plan” to guide clinical decision making. Plans may vary between institutions to best cater to different practice environments. Some of the typical contraindications to ECMO include but are not limited to advanced age, active malignancy, severe aortic insufficiency, severe peripheral vascular disease, inability to tolerate therapeutic anticoagulation, and multiple end-organ dysfunction [6].

ECMO is a labor and resource-intensive therapy that requires multidisciplinary input and extensive training. Its use should be limited to those where cardiac recovery is expected or as a bridge to a more definitive therapy such as transplantation or ventricular assist devices (VADs) [7]. These strict criteria limit the number of ECMO candidates in attempts to ensure the best outcomes from an invasive and resource intensive intervention.

These considerations also come in to play when evaluating patients for short course ECMO support or “prophylactic” ECMO that are undergoing high-risk procedures in the CCL. Patients in need of complex coronary revascularization or other endovascular cardiac procedures who are deemed too high risk for cardiac surgery could benefit from ECMO support during their procedure. In some cases, having vascular access established prior to the planned intervention could also save time in the event of decompensation requiring emergent ECMO cannulation. ECMO-supported procedures can provide hemodynamic stability and critical time to treat myocardial ischemia or relieve critical aortic valve stenosis in otherwise unstable patients. The potential use of ECMO during interventional procedures should be discussed with patients during the informed consent process. Likewise, it should also be discussed if a patient is deemed not an ECMO candidate prior to an intervention and the implications of clinical deterioration during the planned procedure. Here, we will review the use of ECMO in the CCL as ECPR, as a rescue for the hemodynamically unstable patient, and as a prophylactic measure to perform high-risk interventions.

## 2. ECPR in the Cardiac Catheterization Lab

In the event of a cardiac arrest in the CCL, ECPR can be utilized, in which patients in refractory arrest are started on V-A ECMO. ECPR was first hypothesized and attempted in the 1990s during a study conducted by Younger et al. that found ECMO could be successful in select patients with reversible disease processes such as respiratory failure or pulmonary emboli [8]. One of the first trials that investigated the use of ECMO for refractory cardiac arrest was the CHEER trial in 2014 [9]. The CHEER trial was able to significantly determine that patients with refractory cardiac arrest, which they defined as >30 min of CPR, benefit from ECMO initiation, with 54% of patients who underwent ECMO and hypothermic therapy after arrest able to achieve the return of spontaneous circulation (ROSC) and be weaned from ECMO [9]. More recently, the use of ECPR in the CCL has grown with a recent study citing its use for refractory CS or cardiac arrest (CA) demonstrating acceptable survival in a group that otherwise has an extremely high mortality (CA: 44% vs. CS: 52%, *p* = 0.41) [10]. Further investigation by the Mayo Clinic with their experience found a 60% 30-day survival in the 25 patients that underwent ECMO cannulization for CS or CA in the CCL [11]. Interestingly, the mean Acute Physiology and Chronic Health Evaluation (Apache) IV score was 113, which correlated to a predicted cohort mortality of 73%, which was higher than that observed. Overall, 80% of the survivors were eventually weaned from ECMO, and three patients required bridging to VAD or transplant [11].

### 2.1. High-Risk Coronary Artery Disease

The use of ECMO-supported percutaneous coronary interventions (PCIs) provides complete hemodynamic support in patients with ongoing or potential hemodynamic collapse. In comparison to IABP or coaxial devices, ECMO provides right ventricular offloading and oxygenates blood. Griffeoen et al. evaluated the use of V-A ECMO in patients that were high risk for PCI and not surgical candidates for revascularization [12]. Overall, 13/14 patients underwent successful revascularization with periprocedure ECMO support, and 12/14 survived 60 days. The mean duration of support was 151 min, and only 1 patient required prolonged ECMO support (2 days) [12]. Additional small institutional series describe the use of ECMO for elective high-risk PCI with most patients being able to be weaned off ECMO prior to the end of the procedure and no in-hospital death [13,14]. Many of the patients in these studies were also deemed not surgical revascularization candidates.

Although there has yet to be a large-scale study on the use of ECMO in PCI, these early results are promising that such high-risk PCI procedures can be performed with limited morbidity or mortality in appropriately selected patients. Brscic et al. carried out prophylactic ECMO cannulation for 27 high-risk patients undergoing PCI or structural heart procedures with numerous comorbidities. There were 4 (8%) deaths reported in the 6-month follow-up period; however, none were secondary to cardiac causes [15]. The investigators believed ECMO allowed them to conduct more complex procedures in patients that may not have otherwise tolerated it, and they could have suffered from intraprocedural cardiac events and arrest [15]. Ungureanu et al. explored the use of prophylactic V-A ECMO in high-risk patients deemed inoperable and admitted for heart failure with severe coronary artery disease [16]. The study was small, including nine patients, but they observed only one mortality within 30 days of intervention and two deaths within the 3-year follow-up interval [16]. A recent study from Chile had similar outcomes in those undergoing high-risk PCI or transcatheter aortic valve replacement (TAVR) deemed poor surgical candidates but used a modified, low-cost ECMO device with a cost reduction of 72% [17]. Further larger studies and trials are needed to help better define patient selection and determine if the benefits of prophylactic ECMO use in the CCL outweigh the risks in patients previously deemed “too sick” for intervention.

The use of ECMO in these settings is not without risk. While it may allow for high-risk procedures to be performed, the inherent risks of ECMO cannulation remain. Bai et al. explored these risks in a small study with 36 high-risk patients that underwent ECMO-supported PCI. There were no intra-operative deaths reported, and post-procedurally, there was one death (2.8%), one stroke (2.8%), two instances of lower extremity ischemia (5.6%), one deep vein thrombosis (2.8%), two cannulation hematomas (5.6%), and notably five bleeding events requiring transfusion (13.9%) [18]. The only death reported in the study was related to bacteremia and subsequent worsening sepsis during that hospitalization [18]. The reports of limb ischemia, cannulation site injuries, and post-procedural need for dialysis after ECMO support have also been previously reported [11,12,13]. Although reports published thus far appear to have acceptable survival, one must also consider the potential complications of ECMO when deciding to use such technology for a patient in the CCL.

### 2.2. Structural Aortic Valve Disease

The use of ECMO either prophylactically or as a rescue device in structural heart interventions has been described. It has been best reported with TAVR in “high-risk” patients. The definition of the high-risk patient varies between studies, but it broadly includes those with severe decompensated heart failure, borderline cardiovascular collapse, hemodynamic instability either with balloon aortic valvuloplasty or due to arrhythmias, or severe aortic valvular disease that would otherwise preclude them from operative intervention [19,20]. Emergent indications for ECMO in TAVR often include ventricular or aortic annulus rupture, cardiac arrest, hemodynamic deterioration, or coronary obstruction. When these situations are encountered, large bore vascular access is usually already obtained, and the TAVR arterial access and venous temporary pacing sheaths can be quickly replaced with cannulas.

When comparing the prophylactic use of ECMO to emergent cannulation in TAVR (ventricular perforation, hemodynamic deterioration), prophylactic cannulation had a higher procedural success rate (100% vs. 44%) and a lower 30-day mortality (0% vs. 44%) despite a higher median logistic EuroScore in the prophylactic cohort (30% vs. 15%) [19]. Another study from Australia analyzed the outcomes of TAVR with or without ECMO support. Of the 100 TAVR patients, 11 utilized ECMO. Survival was similar between groups with no deaths in the prophylactic ECMO use, one with rescue ECMO use, and two non-ECMO patients. Those requiring ECMO were more likely to develop acute renal failure (36% vs. 8%); however, the majority were from the emergent cannulation cohort [20]. Further, a more recent systematic review included nine studies with an overall 4% ECMO utilization rate in the early TAVR experience with a 29.8% short-term (in-hospital, 30-day) and 52.4% 1-year mortality [21].

### 2.3. Pulmonary Embolism and “Clot in Transit” Sequelae

There is growing interest for the percutaneous interventions of pulmonary emboli (PE) and clots in transit. The use of ECMO in this setting is to provide hemodynamic support and oxygenation, as there is often significant loss of left ventricular (LV) preload, a marked increase in right ventricular (RV) afterload, reduced stroke volume, impaired systemic perfusion, and eventual cardiopulmonary collapse. While ECMO is still only used for 0.2% of PEs, previous work suggested a possible improvement in survival with ECMO use in high-risk PE [22]. Furthermore, ECMO can provide systemic perfusion while procedures like thrombectomy are performed [23,24]. For patients that cannot receive thrombolytic treatment, case reports and retrospective studies of aspiration embolectomy on ECMO have been proven as potential alternatives [25,26].

Several studies have assessed the best management strategies for massive PEs requiring ECMO support. One such study compared ECMO alone, ECMO plus fibrinolysis, and ECMO combined with surgical embolectomy [27]. The results showed that ECMO plus surgical embolectomy yielded the lowest 30-day mortality rate (29%) compared to ECMO alone (78%) or ECMO plus fibrinolytics (77%) [27]. Given that patients with massive or submassive PE requiring surgery are typically the most critically ill, it is notable that surgical embolectomy combined with ECMO not only showed success but outperformed the other treatment methods. Previous studies also showed operative mortality as low as 4–6% for embolectomy, although not all patients were supported with ECMO pre-operatively [28,29]. A major limitation though is in the selection bias, since not all surgical candidates may have been the highest risk.

For patients who have an absolute contraindication to thrombolytic therapy, aspiration embolectomy in the CCL under ECMO support could prove to be an alternative. Patel et al. describes a neurosurgical patient that was acutely unstable after the discovery of a large pulmonary embolus. After ECMO cannulation, they were able to successfully remove a large portion of the PE burden, resolving enough for resolution of the obstructive shock [25]. Another case series of nine patients on ECMO undergoing percutaneous thrombectomy (six had a previous cardiac arrest) had a 22% 90-day mortality [30]. A larger institutional series from the University Hospital Zurich was published in which critically ill patients with contraindication to thrombolytics or failed thrombolytic therapy underwent aspiration embolectomy with ECMO support or with ECMO standby [26]. Of the 15 patients that underwent aspiration embolectomy, 8 were cannulated on ECMO prior to intervention. Three patients without initial ECMO support experienced a cardiac arrest (two ultimately were cannulated for ECMO). ECMO was successfully weaned in all patients over a mean of 5.4 days. There was on periprocedural death and an overall 33% 30-day mortality [26]. Despite the small sample size, the study highlights that it might not be safe to perform these procedures without at least ECMO standby, and consideration should be made to cannulate these patients prior to the embolectomy if there is concern for intraprocedural hemodynamic instability.

### 2.4. Catheter Ablation

The use of ECMO to support perfusion during episodes of refractory tachyarrhythmias, particularly in the context of hemodynamic instability and electrical storm, has been previously shown to have potential benefits [31,32]. In this context, ECMO can often be a bridge to definitive ablative therapies. In those with end-stage heart failure, ECMO-supported catheter ablation of unstable ventricular tachycardia (VT) can also be used as a bridge to VAD or transplantation [33].

Moreover, there are studies utilizing ECMO as an adjunct to catheter ablation in the CCL given that some ablations necessitate continuous VT, which can result in hemodynamic instability. Initial investigations suggest that like other short-term, V-A ECMO-supported procedures, catheter ablation with ECMO support tends to be safe and effective with a single institutional study (n = 21) reporting 95% ablation success and 100% survival at 1 year follow-up [34]. In a different study that looked at patients who had acute hemodynamic instability during VT ablation (without ECMO support), there was a 38% all-cause mortality at 1 year [35]. The use of ECMO in patients with unstable ventricular arrhythmias also appears to facilitate accurate mapping, as polymorphic VTs were still able to be induced at the conclusion of the procedure in 8/15 (53%) patients not supported on ECMO and 2/31 (6%) of those supported with ECMO [36]. The ECMO-supported patients also had less implantable cardioverter–defibrillator intervention (shock or anti-tachycardia pacing) at long-term follow-up (19% vs. 42%) suggesting an improvement in the quality of life [36].

## 3. Discussion

The use of periprocedural ECMO in the CCL has opened a new possibility to expanding catheter-based coronary, structural heart, and electrophysiological interventions to patients that were previously deemed too high risk for such interventions. However, most of the data regarding its use thus far have been from single centers with small sample sizes.

The use of ECMO in the CCL currently remains limited to either planned intraprocedural support in patients at high-risk of hemodynamic collapse or as a rescue device in patients experiencing an intraprocedural cardiac arrest or refractory shock. Unlike other scenarios, vascular access is often already obtained in patients necessitating emergent ECMO cannulation. This is a major advantage, as it expedites cannulation and decreases tissue hypoxia and malperfusion. Furthermore, decreasing the time between the cardiac event and cannulation of the patient has been linked to improved outcomes and better neurologic function after the event [37]. That said, prophylactic CCL cannulation has been shown to have improved outcomes, [15,16,19,20,34], and providers should balance the risk of upfront intraprocedural ECMO versus the realistic potential of a crashing patient that will ultimately still require ECMO more emergently.

Consideration for prophylactic ECMO should be given to patients undergoing a “high-risk” intervention. Although “high-risk” remains poorly defined, patients with decompensated heart failure, hemodynamic instability, ventricular dysfunction, pulmonary hypertension, and unstable arrhythmias should all be considered for an ECMO-supported procedure. Additional regard needs to be taken regarding the complexity of the proposed intervention and whether periods of hemodynamic instability that could result in cardio-metabolic shock are expected.

When deciding who would be a candidate for ECMO, thought should be taken as to the end outcome and long-term goals for that patient. What is the life expectancy of the patient? How long has the patient had intraprocedural hemodynamic compromise? Is the patient likely to recover and be decannulated? If not, will the patient be a candidate for a transplant or VAD? For elective interventions such as VT ablation, this decision is often easier, as ECMO is used more for intraprocedural hemodynamic support. In patients arriving to the CCL decompensated or with unfavorable anatomy or physiology, the decision becomes more difficult, as there needs to be consideration for the next step after the patient is cannulated. In the setting of the coding patient, ECPR should be considered as an adjunct to advanced cardiac life support (ACLS) therapies. It would be advantageous to have an understanding if a patient is an ECPR candidate prior to any planned CCL intervention.

Discussion regarding a patient’s candidacy for ECMO in the CCL should be had with the entire Heart Team. This should include heart-failure cardiology, interventional cardiology, cardiothoracic surgery, perfusionists, and intensivists. Early involvement of the entire Heart Team is crucial for adequate planning and preparation for taking on the care of these critically ill patients and determining if they are a candidate for advanced therapies. This also ensures that the education on the risk, benefit, contraindications, and complications are consistent amongst the evaluation of different patients.

These discussions should also be carried out with patient and family prior to any intervention to best align with the treatment goals of the patient. Having specialists who are knowledgeable of ECMO and the associated complex care can also aid with patient and family education when it comes to making difficult decisions regarding their care goals. Ideally, these discussions are had at time of procedural consent to offer a transparent explanation to patients of their ECMO candidacy.

For patients requiring ECMO, multidisciplinary care coordination is necessary. Having a highly trained Heart Team and support stuff is critical. At the bedside, qualified intensivists, nursing staff, and perfusionists are crucial to provide complex minute-by-minute decision making 24 h a day. The involvement of vascular surgery is also essential to assess distal limb perfusion and provide support for vascular access repairs at time of decannulation.

It takes a village to keep these patients alive and on the road to recovery, and having the necessary infrastructure in place prior to implementation of an ECMO service is critical to success. Developing predetermined anticoagulation strategies, weaning protocols and designated teams—like the pulmonary embolism response team (PERT) and stroke team—could prove advantageous for patient outcomes and improve the capability for prospective study.

## 4. Conclusions

Extracorporeal membrane oxygenation, though costly and resource-intensive, is an emerging resource in the cardiac catheterization lab. Current limited data suggest it provides hemodynamic stability to patients undergoing high-risk coronary, structural heart, and electrophysiological catheter-based interventions. It has also been used to help support high-risk patients—who may otherwise be considered ineligible—through such interventions. ECMO has also been described as a rescue device in the setting of an intraprocedural cardiac arrest. Regardless, consideration of the “next step” and a patient’s likelihood to be weaned from ECMO or candidacy for advanced cardiac therapies is required at time of cannulation. Although the current use of ECMO in the cardiac catheterization lab is promising, future multicenter, prospective studies are still needed to improve patient selection and better understand how to utilize the technology.
